# Preventive effects of *Mycobacterium tuberculosis* DNA vaccines on the mouse model with latent tuberculosis infection

**DOI:** 10.3389/fimmu.2023.1110843

**Published:** 2023-02-13

**Authors:** Yan Liang, Xiaoping Li, Yourong Yang, Li Xiao, Yumei Liang, Jie Mi, Yong Xue, Wenping Gong, Lan Wang, Jie Wang, Junxian Zhang, Yingchang Shi, Bizhen Peng, Xiaoyang Chen, Weiguo Zhao, Xueqiong Wu

**Affiliations:** ^1^ Tuberculosis Prevention and Control Key Laboratory, Beijing Key Laboratory of New Techniques of Tuberculosis Diagnosis and Treatment, Senior Department of Tuberculosis, the Eighth Medical Center of PLA General Hospital, Beijing, China; ^2^ Department of Respiration, Hengdong People’s Hospital, Hengyang, China; ^3^ Department of Respiration, the Eighth Medical Center of PLA General Hospital, Beijing, China; ^4^ Department of Pathology, the Eighth Medical Center of PLA General Hospital, Beijing, China

**Keywords:** DNA vaccine, Mycobacterium tuberculosis, latency-associated antigen, latent tuberculosis infection, preventive effects

## Abstract

**Background:**

About a quarter of the world’s population with latent tuberculosis infection (LTBI) are the main source of active tuberculosis. Bacillus Calmette Guerin (BCG) cannot effectively control LTBI individuals from developing diseases. Latency-related antigens can induce T lymphocytes of LTBI individuals to produce higher IFN-γ levels than tuberculosis patients and normal subjects. Herein, we firstly compared the effects of *M. tuberculosis* (MTB) *ag85ab* and 7 latent DNA vaccines on clearing latent MTB and preventing its activation in the mouse LTBI model.

**Methods:**

A mouse LTBI model was established, and then immunized respectively with PBS, pVAX1 vector, Vaccae vaccine, *ag85ab* DNA and 7 kinds of latent DNAs (including *rv1733c*, *rv2660c*, *rv1813c*, *rv2029c*, *rv2628*, *rv2659c* and *rv3407*) for three times. The mice with LTBI were injected with hydroprednisone to activate the latent MTB. Then, the mice were sacrificed for the bacterial count, histopathological examination, and immunological evaluation.

**Results:**

Using chemotherapy made the MTB latent in the infected mice, and then using hormone treatment reactivated the latent MTB, indicating that the mouse LTBI model was successfully established. After the mouse LTBI model was immunized with the vaccines, the lung colony-forming units (CFUs) and lesion degree of mice in all vaccines group were significantly decreased than those in the PBS group and vector group (*P*<0.0001, *P*<0.05). These vaccines could induce antigen-specific cellular immune responses. The number of IFN-γ effector T cells spots secreted by spleen lymphocytes in the *ag85ab* DNA group was significantly increased than those in the control groups (*P*<0.05). In the splenocyte culture supernatant, IFN-γ and IL-2 levels in the *ag85ab*, *rv2029c*, and *rv2659c* DNA groups significantly increased (*P*<0.05), and IL-17A levels in *ag85ab* and *rv2659c* DNA groups also significantly increased (*P*<0.05). Compared with the PBS and vector groups, the proportion of CD4^+^CD25^+^FOXP3^+^ regulatory T cells in spleen lymphocytes of *ag85ab*, *rv2660c*, *rv2029c*, and *rv3407* DNA groups were significantly reduced (*P*<0.05).

**Conclusions:**

MTB *ag85ab* and 7 kinds of latent DNA vaccines showed immune preventive efficacies on a mouse model of LTBI, especially the *rv2659c*, and *rv1733c* DNA. Our findings will provide candidates for the development of new multi-stage vaccines against TB.

## Introduction

Tuberculosis (TB) is still a serious global infectious disease. There were more than 10.60 million new TB cases and 1.6 million deaths, and about a quarter of the world’s population was infected with *Mycobacterium tuberculosis* (MTB) (1). Of these MTB-infected individuals, 90–95% of them have a long-term state of latent tuberculosis infection (LTBI), but 5–10% of them will develop to active tuberculosis (2, 3). LTBI is a special condition in which an individual has been infected with MTB but has not yet developed active TB (ATB), which is characterized by MTB antigen-specific immune responses without the clinical manifestations and imaging changes of ATB. Epidemiological surveys have found that about 85%-90% of the new cases of active pulmonary TB progressed from LTBI (4). Therefore, the prevention and control of LTBI is the key to control of TB. So far, Bacillus Calmette Guerin (BCG) is the only licensed pre-exposure anti-TB vaccine in the world, but it cannot effectively control the disease development of LTBI individuals (5). In China, *Mycobacterium vaccae* inactivated vaccine (Vaccae vaccine) has been approved as a commercial vaccine for tuberculosis immunotherapy and LTBI preventive treatment. The phase III clinical trial of Vaccae vaccine used in healthy adults with strong positive purified protein derivative (PPD) skin test (≥15mm) showed that its protection rate was 54.7% (6). A phase IIb clinical trial of M72/AS01_E_ subunit vaccine used in QuantiFERON-TB Gold In-Tube assay (QFT-Gold)-positive adults showed that the protection rates after 1 year, 2 years, 3 years and the total protection rate were 27.4%, 55.2%, 60.2%, and 49.7% respectively (7). However, the protection rate of the H4/IC31 subunit vaccine in the phase II clinical trial was only 30.5% for the continuous QFT-positive population, which was lower than 45.4% of the BCG vaccine (8). Obviously, the protection rates of current vaccines entering clinical trials for the LTBI population were not ideal. New and more effective vaccines are needed to protect LTBI individuals from endogenous reactivation of latent MTB.

During latent TB infection, antigens expressed by dormant MTB may be candidate immune markers for protection from MTB (9). Many studies have shown that up-regulated DosR regulon antigens, for example, *rv1733c*, *rv1813c*, *rv2029c*, *rv2628*, *rv2659c*, and *rv2660c*, could strongly induce T cells of LTBI individuals to secrete IFN-γ under the conditions of hypoxia or nutrient deprivation during the dormant stage of MTB infection, suggesting that the effective recognition of these antigens by T cells will help to control latent MTB infection (10–16). Rv1733c is a conservative trans-membrane protein, which is highly expressed during MTB hypoxia dormancy and can be recognized by peripheral blood T lymphocytes of LTBI individuals, thus inducing T lymphocytes to secrete high levels of IFN-γ (10). Plasmid pcDNA-*rv1733c* DNA could produced specific humoral and cellular immune responses in mice (11). Rv2029c is a 6-phosphofructokinase 2 (PfkB) that is an important rate-limiting enzyme in glucose metabolism. In the hypoxic and nutrient deficient environment of tuberculous granuloma, it plays a key physiological regulation role in the glycolysis process of MTB, meeting the nutritional requirements of the latent MTB survival and maintaining their growth (17). Rv1813c cotranscribed with the downstream gene *rv1812c* is up-regulated during dormancy, which can regulate all aspects of MTB virulence *in vivo* and induce immune responses to infection (11). Rv2628 may be a hypothetical protein regulated by DosR, and its function is poorly understood. Rv2029c and Rv2628 can also be highly recognized by peripheral blood T lymphocytes of LTBI individuals, and mainly induce CD4^+^ central memory T cells (CD45RO^+^CD27^+^) to secrete high levels of IFN-γ, which may contribute to the clearance of latent MTB or inhibit its reactivation (14, 18, 19). Rv2659c and Rv2660c are the starvation-related proteins encoded by the genes within the RD11 region of MTB. Rv2659c is a probable PhiRv2 prophage integrase, and Rv2660c is a hypothetical protein. Rv2660c and Rv2659c can also induce IFN-γ in the LTBI individuals (13, 16). Rv3407 protein is the same name as antitoxinVapB47 and a molecular weight of 11 kDa (20), is a specific protein produced during transformation of MTB from latency to reactivation, regulating nutrition and metabolism (21, 22).

Current studies have shown that latency-associated antigen is usually used as a component of preventive or therapeutic vaccines. For example, antigen Rv1813 was a component of the MTB subunit vaccine (ID93) and the MTB fusion protein (ID83), which induced a Th1-type immune response and protected mice against MTB (23, 24). The subunit vaccine H56 containing the antigen Rv2660c could protect nonhuman primates from both pre- and post-MTB infection (25, 26). Synthetic peptide of latent antigen Rv1733c with CpG adjuvant significantly reduced the pulmonary bacterial loads in the mouse model with a pre- and post-MTB infection (27), and the subunit vaccine H83 containing the antigen Rv1733c could significantly decrease the bacterial load in an LTBI rabbit model (28). A DNA vaccine expressing latency-associated antigen Rv3407 induced Th1-type cellular immune responses and reduced lung bacterial loads (29). A recombinant BCG vaccine expressing three antigens Rv2659c, Rv3407, and Rv1733c could improve protection from MTB for a long time (30). Thus, antigens expressed by dormant MTB are potential vaccine candidates (9). Our previous studies found that 7 DNA vaccines expressing Rv1733c, Rv1813c, Rv2628, Rv2029c, Rv2659c, Rv2660c, and Rv3407 had some immunotherapy effect on the MTB endogenous reactivation or MTB infection mouse models (29, 31). Although these latent antigens as vaccine components were effective in preventing MTB infection or LTBI reactivation, it is not clear which latency-antigens as vaccine components are more effective in clearing latent MTB and providing better protection against LTBI.

Therefore, in this study, using chemotherapy made the MTB latent in the infected mice, and then using hormone treatment reactivated the latent MTB to establish a mouse LTBI model. For the first time, the Vaccae vaccine, MTB *ag85ab*, and these 7 latent DNA vaccines were evaluated the effects on clearing latent MTB, and preventing its reactivation and reducing the degree of pulmonary lesions in the mouse LTBI model, to lay a foundation for the construction of multi-antigen and multi-stage vaccines that can provide more comprehensive protection against MTB infection in the future.

## Materials and methods

### Ethics statement

All animals experiments were approved by the Animal Ethical Committee of the Eighth Medical Center of the Chinese PLA General Hospital, and the care of mice met the standards of the Regulations on the Management of Laboratory Animals formulated by the State Science and Technology Commission of China. All animal experiments were performed in a qualified Level-II negative pressure bio-safety laboratory of the Eighth Medical Center of Chinese PLA General Hospital.

### Mice

One hundred fifty-eight BALB/c female mice aged 6~8 weeks without specific pathogens were purchased from Beijing Vital River Laboratory Animal Technology Limited Company, China, and kept in a qualified Level-II negative pressure bio-safety laboratory of the Eighth Medical Center of PLA General Hospital, Beijing, China.

### MTB strain and its infection

MTB H_37_Rv was provided by National Institutes for Food and Drug Control, Beijing, China. The MTB H_37_Rv strain was cultured on a Lowenstein-Jensen medium (Zhuhai Baso Biotechnology Co., LTD., Guangdong province, China) at 37°C for three weeks. MTB H_37_Rv colonies were scraped, weighed, and homogenized in 0.5% Tween 80-saline to prepare 0.1mg/ml MTB H_37_Rv suspension, which was stored at -20°C. Before use, the frozen MTB H_37_Rv suspension was thawed, and then further continuously diluted 10-fold. 100 μl of each dilution was added to Lowenstein-Jensen plates twice and cultured at 37°C for 4 weeks. The bacterial colonies on each plate were counted. The 0.1mg/ml bacterial suspension contained 6.4×10^4^ colony-forming units (CFUs)/ml, 0.4 ml was injected into the tail vein of each mouse. In this study, 2.56×10^4^ CFUs of MTB H_37_Rv was challenged into the tail vein of each mouse.

### Preparation of DNA vaccines and their recombinant proteins

The construction of MTB *ag85ab, rv1733c*, *rv2660c*, *rv1813c*, *rv2628*, *rv2029c*, *rv2659c* and *rv3407* DNA vaccines was described in our previous studies (28, 30, 32). DNA vaccines were purified using the EndoFree Plasmid Purification Kit (Qiagen, Hilden, Germany).

Five MTB recombinant plasmids of Ag85AB, Rv1813-, Rv2628-, Rv2029c-, and Rv2660c/Rv2659c-pET30a were constructed, and expression and purification of their proteins were described in our previous studies (30–32). MTB recombinant Rv3407 and Rv2029c proteins were purified by Guangdong TB Healthcare Biotechnology Co., LTD, Guangdong, China. Unfortunately, the expression of recombinant Rv1733c protein was unsuccessful.

### Preparation and treatment of the mouse model with latent tuberculosis infection

**Figure 1:**
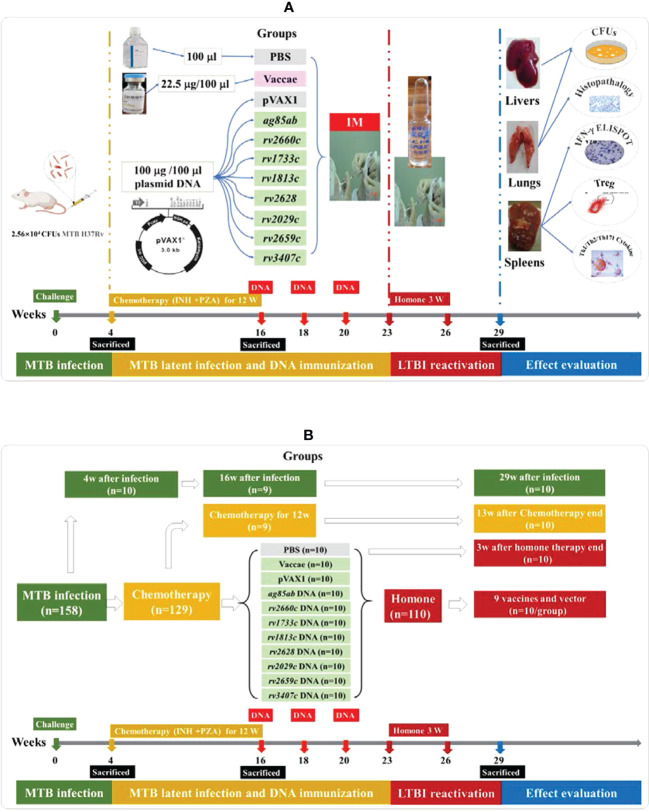
The experimental schedule and grouping diagram of this study. **(A)** the experimental schedule diagram of this study; **(B)** the grouping diagram of this study.

The experimental schedule and grouping diagram of this study were shown in **Figure 1**. The mouse model of LTBI was prepared through the following three stages: Firstly, 158 BALB/c female mice aged 6~8 w were intravenously infected with 25600 CFUs of MTB H_37_Rv. As controls, 29 mice untreated infected with MTB were used as controls for tuberculosis model. Secondly, at 4w after infection, 129 infected mice were treated with Isoniazid (INH) (0.12 g/L, Tianjin Lisheng Pharmaceutical Co., LTD, Tianjin, China) and Pyrazinamide (PZA) (8g/L, Shenyang Hongqi Pharmaceutical Co., LTD, Shenyang, China) for 12w. At 16 weeks after infection (i.e. after chemotherapy for 12w), 110 mice were randomly divided into eleven groups and given intramuscular immunization for three times with an intervals of 2 w as follows: (1) PBS as a negative control (100 μl); (2) vector pVAX1 DNA as a negative control (100 μg/100 μl); (3) Vaccae vaccine (22.5 μg/100 μl, Anhui Zhifei Longcom Biologic Pharmacy, Anhui, China) as a positive control, approved by the National Medical Products Administration in China for the preventive treatment of individuals with LTBI in 2021; (4) *ag85ab* DNA (100 μg/100 μl, Guangzhou Baiyunshan Baidi Biological Medicine Co., LTD, Guangzhou, China);(5) *rv2660c* DNA (100 μg/100 μl); (6) *rv1733c* DNA (100 μg/100 μl); (7) *rv1813c* DNA (100 μg/100 μl); (8) *rv2628* DNA (100 μg/100 μl); (9) *rv2029c* DNA (100 μg/100 μl); (10) *rv2659c* DNA (100 μg/100 μl); (11) *rv3407* DNA (100 μg/100 μl). Thirdly, at 3 w after the end of preventive immunotherapy, 110 mice were intramuscularly injected with hydroprednisone injection (0.5 mg/mouse, Jiangxi National Medicine Co. LTD, Jiangxi, China) three times a week for 3 w. After the end of chemotherapy for 12w, 19 mice were untreated with hydroprednisone injection as controls.

### Bacterial counts in lung and liver tissue

The left lung and the upper half of the liver from each mouse were taken at six different time points as follows: 4 w, 16 w, and 29 w after infection, at the end of chemotherapy for 12w, and at 13w after the end of chemotherapy, at 3 w after the end of hormone therapy, homogenized with 3 ml of sterile saline, and then digested with an equal volume of 4% NaOH for 30 minutes. The tissue suspensions of lungs and livers were diluted respectively by 10-fold series (100 μL lung tissue suspension added 900μL saline), and 100 μL of tissue suspensions from each dilution (the tissue suspensions digested by 4% NaOH, 10^-1^, 10^-2^) were inoculated respectively on Lowenstein-Jensen plates in two copies and cultured at 37°C for 4 w. Enumerated the MTB colonies on each plate. The bacterial colony number of the left lung obtained was converted into that of the whole lung according to the weight of the left lung and the whole lung, and the bacterial colony number of the upper half of the liver obtained was converted into that of the whole liver according to the weight of the upper half of the liver and the whole liver. The results were expressed as CFUs of each lung.

### Pulmonary histopathological examination

The right lung of each mouse was fixed in 10% buffered formalin and paraffin-embedded. The prepared paraffin-embedded lung tissue sections were stained with hematoxylin and eosin by Lycra auto dyeing machine (Lycra, German). The images of all tissue sections from the right lung were obtained with a Huahai pathological diagnosis system (Huahai medical information company, Xi’an, China), and then were analyzed histopathological changes and the percentage of lung lesion area and calculated the average area of lung tissue lesions in each group by an experienced pathologist.

### IFN-γ enzyme-linked immunospot assay

The splenocytes were isolated from five mice each group, plated in duplicate (3×10^5^ cells/well), and incubated at 37°C for 24h with medium 1640 (as a negative control), recombinant protein Ag85AB (at the final concentration of 20 μg/ml) or recombinant Rv1813, Rv2029c, Rv2628, Rv3407, and Rv2660c/Rv2659c mixed proteins (each protein at the final concentration of 20 μg/ml, not including recombinant Rv1733c protein) or with phytohemagglutinin (PHA, Sigma, USA, at the final concentration of 20 μg/ml, as a positive control). Twenty-four hours later, the number of spot-forming cells (SFCs) was measured using a Mouse IFN-γ ELISPOT ^PLUS^ assay kit (Cat. No. 3321-4APT-2, Mabtech AB, Nacka Strand, Sweden) following the kit’s instructions. SFCs in each well were automatically enumerated using a CTL-ImmunoSpot^®^ S5 Micro Analyzer (Cellular Technology, Cleveland, Ohio, USA). The different values between the stimulation wells and the negative wells in each group were calculated as the average number of SFC.

### Analysis of CD4^+^CD25^+^FOXP3^+^ T cell subsets

The spleen cells were isolated from five mice each group, 100 µl containing 3×10^6^ splenocytes was transferred into a 12×75 mm FACS tube containing FITC Rat Anti-Mouse CD4 (2 µl, Cat. No. 553046, Clone: RM4.5, BD Biosciences, San Jose, Ca, USA; CD4-FITC, BD Biosciences), and APC Rat Anti-Mouse CD25 (5 µl, Cat. No. 557192, Clone: PC61, BD Biosciences, San Jose, Ca, USA). The FACS tubes were incubated at 4°C in the dark for 30 min. 2 ml of precooled PBS was added to each tube, then centrifuged at 4°C and 1200 rpm for 5 min. The supernatant was sucked away and 1ml of 1×fixation/permeabilization solution (Foxp3/Transcription factor staining buffer set Cat. No. 00-5523, eBioscience, San Diego, Ca, USA) was pipetted into the tube and incubated at 4°C in the dark for 40 min. 2 ml of permeabilization buffer (FoxP3/Transcription factor staining buffer set Cat. No. 00-5523, eBioscience, San Diego, Ca, USA) was pipetted into the tube, then centrifugation at 4°C and 1200 rpm for 5 min. After the supernatant was sucked away, each tube received 2 ml permeabilization buffer and was then centrifuged at 4°C and 1200 rpm for 5 min. After the supernatant was sucked away, 2μl normal rat serum was pipetted into the tube and incubated at room temperature in the dark for 15 min. PE Anti-Mouse/Rat FoxP3 (5 µl, Cat. No. 12-4776-42, Clone: FJK-16s, eBioscience, San Diego, Ca, USA), APC Rat IgG1 λ Isotype control (2.5 µl, Cat. No. 550884, BD Biosciences, San Jose, Ca, USA), PE Rat IgG2a κ isotype (2.5 µl, Cat. No. 12-4321, eBiosciences, San Diego, Ca, USA) were pipetted into the tube, and incubated at 4°C in the dark for 40 min. 2 ml of permeabilization buffer and was added into the tube, then centrifuged at 4°C and 1200 rpm for 5 min and the supernatant was aspirated, then wash again. 2 ml precooled PBS was added into each tube, then centrifuged at 4°C and 1200 rpm for 5 min. After the supernatant was aspirated, 500 µl PBS was pipetted and analyzed within 1 h by FACS Canto II flow cytometry (BD Pharmingen) and FACS Diva software. The representing FACS graphs with the gating method were described in our previous study (33).

### Th1/Th2/Th17 cytokine analyses

The splenocytes were prepared by the above methods. 100µl splenocytes (3×10^6^/ml) with 50µl recombinant protein Ag85AB (the final concentration of 20 μg/ml) or recombinant Rv1813, Rv2029c, Rv2628, Rv3407, and Rv2660c/Rv2659c mixed proteins (the final concentration of 20 μg/ml, not including recombinant Rv1733c protein) or PHA (Sigma, USA, the final concentration of 20 μg/ml, as a positive control) were cultured in 96-well cell culture plate (Corning, USA) at 37°C for 48 h. Then, the culture solution was then sucked into a new 1.5 ml tube and centrifugated at 5000 rpm for 3 min. Finally, the supernatant was gently sucked into another new 1.5 ml tube, and the levels of IFN-γ, interleukin -2 (IL-2), tumor necrosis factor (TNF), IL-4, IL-6, IL-10 and IL-17A were measured using a Mouse Th1/Th2/Th17 Cytokine Kit (Cat. No. 560485, BD Biosciences, San Jose, CA, USA) according to the manufacturer’s instructions, and determined by FACS Canto II flow cytometry (BD Pharmingen) and analyzed using BD FCAP Array v3.0 software.

### Statistical analyses

The data are expressed as the mean ± standard deviation. Statistical analyses were conducted using a one-way ANOVA followed by a Dunnett’s multiple comparison test between the groups, or a Kruskal-Wallis test followed by a Student-Newman-Keuls test between the groups. A *P*-value of <0.05 was considered statistically significant.

## Results

### Preparation of the mouse model with latent tuberculosis infection

The mice were infected intravenously with 25600 CFUs of MTB H37Rv. At 4w after infection, 129 infected mice were treated with INH and PZA for 12 weeks to induce MTB latency. The mice without receiving any vaccine treatment were used to first validate the MTB into latency after chemotherapy, and then validate the reactivation of the latent MTB after hormone treatment.

**Figure 2:**
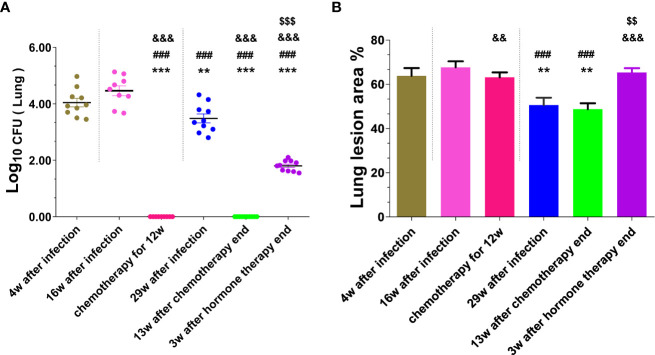
The number of viable MTB and histopathological changes in the lung tissues were obtained from mice at different stages of mouse LTBI model preparation. **(A)** Lung CFUs; **(B)** Lung lesion area%. Comparison with 4w after infection group: ^**^ *P* < 0.01, ^***^ *P* < 0.0001; Comparison with 16w after infection group: ^###^ *P* < 0.0001; Comparison with 29w after infection group: ^&&^ *P* < 0.01, ^&&&^ *P* < 0.0001; Comparison with chemotherapy for 12w group or 13w after chemotherapy end: ^$$^ *P* < 0.01, ^$$$^ *P* < 0.0001.

The mice were sacrificed respectively at 4, 16, and 29 weeks after infection with MTB, and colonies of viable MTB in the lung and liver tissues of each group were measured (shown in **Figure 2A**, **Supplementary Figure 1**), and their pulmonary histopathological changes were examined (shown in **Supplementary Figure 2**, **Figure 2B**). At 4 weeks after infection, the bacterial loads in the lungs and livers of infected mice were 20282 and 7313 CFUs respectively, and the lung tissue presented a large number of lymphocytes, thickened alveolar walls, and extensive lung lesions, indicating that the mice were successfully infected by MTB. At 16 weeks after infection, the lungs and livers in the control group without chemotherapy maintained higher bacterial loads, in the chemotherapy for 12 weeks group had no MTB growth, but which had no significant difference in the area and degree of lung lesions (*P*>0.05), indicating that the chemotherapy inhibited the growth of MTB and made MTB latent, and did not significantly reduce the lung lesions, the mouse infection model has been changed to an LTBI model after chemotherapy. At 29 weeks after infection, the bacterial loads of the lungs and livers of the infected mice in the control group without chemotherapy and hormone treatment decreased spontaneously compared with that at 4 weeks and 16 weeks after infection (*P*<0.01, *P*<0.0001), but there was no bacterial growth in the 13 weeks after the end of the chemotherapy group and the chemotherapy for 12 weeks group, indicating that the chemotherapy made MTB latent in the infected mice, and the latent MTB was still not activated at 13 weeks after the end of the chemotherapy in the absence of hormone treatment. However, the lung pathological changes in the 29 weeks after infection group and the 13 weeks after the end of chemotherapy group were significantly less than those in the 4, 16 weeks after infection groups, and the chemotherapy for 12 weeks group, in which the alveolar wall was slightly thickened, with a small number of lymphocyte infiltration, and the lesion area was decreased to varying degrees (*P*<0.01, *P*<0.0001), indicating that the latent MTB in the lung did not reactivate spontaneously and the lung lesions recovered a little. However, the lung of the mice at 3 weeks after the termination of hormone therapy showed a significant recrudescence of bacterial growth (1.84 logs) compared with the chemotherapy group (0 CFU) (*P*<0.0001), and their lung histopathological changes were also significantly aggravated (*P*<0.01, *P*<0.0001), indicating that the hormone therapy reactivated the latent MTB in the lungs and induced inflammatory response and inflammatory injury again. But the resurgence of latent MTB was not observed in the livers of all groups after hormone therapy (**Supplementary Figure 1**). The above results suggest that the LTBI model of lung has been successfully established in this study, and it is reliable to use this model to evaluate the effects of DNA vaccines on inhibiting the resurgence of latent MTB.

### Immunotherapy of the mouse model with latent tuberculosis infection

**Figure 3:**
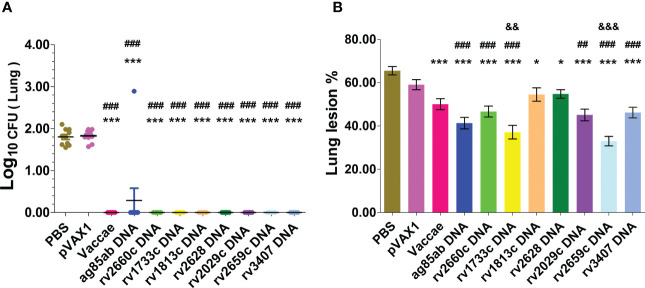
The bacterial lords and histopathological changes in the lung tissues were obtained from mice at 3 w after stopping hydroprednisone injection. **(A)** CFUs of live MTB in mouse lungs; **(B)** Lung lesion area%. Comparison with PBS group: ^*^ *P* < 0.05, ^***^ *P* < 0.0001; Comparison with pVAX1 vector group: ^##^ *P* < 0.01, ^###^ *P* < 0.0001. Comparison with Vaccae vaccine group: ^&&^ *P* < 0.01, ^&&&^ *P* < 0.0001.

After chemotherapy for 12 weeks, the infected mice were treated with PBS, pVAX1 vector DNA, Vaccae vaccine, *ag85ab*, *rv1733c*, *rv1813c*, *rv2029c*, *rv2628*, *rv2659c*, *rv2660c*, and *rv3407* DNA vaccines once every two weeks for three times to evaluate their efficacy on prevention of resurgence. During the experiment, only one mouse of the *rv2660c* DNA group died at 161 d (i.e. 23 w) after the infection. The numbers of liable MTB in the lung and liver tissues after DNA immunotherapy were determined at 3 w after the end of hormone therapy (shown in **Figure3A**). Compared with the PBS group (1.84 ± 0.19 log_10_) and vector group (1.83 ± 0.14 log_10_), there was no bacteria reactivated from the lung of all vaccine groups (*P*<0.0001), except one mouse of the *ag85ab* group (2.89 log_10_).

After the mouse LTBI model was activated with the hormone, representative lung histopathological changes and the percentage of lung lesion area in each group after immunotherapy were shown in **Supplementary Figure 3** and **Figure 3B**. The degree of lung tissue lesion in the vector group was similar to that in the PBS group, in which the lung tissues contained a large number of lymphocytes infiltration, the thickened alveolar wall, and the extensive lung injury. The lung tissues from the Vaccae vaccine, *rv1813c*, and *rv2628* DNA groups contained a medium number of lymphocytes, a medium thickness of the alveolar wall, and significantly reduced lesion areas (*P*<0.0001, *P*<0.05). The lung lesions of the *rv2660c*, *rv2029c*, *rv3407*, *ag85ab*, *rv1733c*, and *rv2659c* DNA groups were mild, with a small number of lymphocyte infiltration, slightly thickened alveolar walls, and significantly reduced lesion areas (*P*<0.01, *P*<0.0001). The lung lesions in the *rv1733c* and *rv2659c* DNA groups were significantly reduced than those in the Vaccae vaccine group, showing a small number of lymphocytes, relatively clear alveolar contour, and normal structure, and the lesion areas were significantly decreased (*P*<0.01, *P*<0.0001), which suggests that the *rv1733c* and *rv2659c* DNA vaccines have better protective effects on the LTBI.

### TB-specific IFN-γ releasing T cell responses

**Figure 4:**
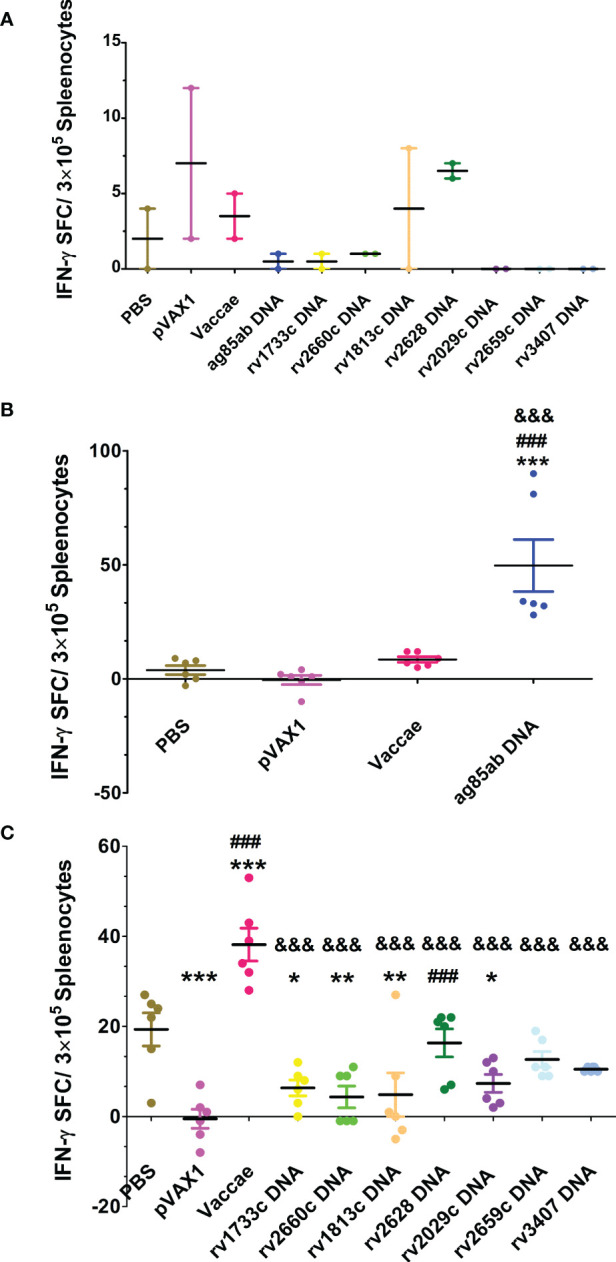
The numbers of spleen lymphocyte spots releasing IFN-γ was detected by ELISPOT assay at 3 w after stopping hydroprednisone injection. **(A)** the numbers of splenocyte spots secreting IFN-γ in unstimulated wells from each group, **(B)** stimulate lymphocyte with Ag85AB protein, **(C)** stimulate lymphocyte with mixed latent proteins. Comparison with PBS group: ^*^ *P* < 0.05, ^**^ *P* < 0.01, ^***^ *P* < 0.0001; Comparison with pVAX1 vector group: ^###^ *P* < 0.0001; Comparison with Vaccae vaccine group: ^&&&^ *P* < 0.0001.

The numbers of spleen lymphocyte spots releasing IFN-γ from each group were determined in response to Ag85AB protein or mixed latent proteins for 24 hours by ELISPOT assay (shown in **Figure 4**). The numbers of T cell spots secreting IFN-γ from most mice except for *rv2628* DNA and pVAX1 groups didn’t significantly increase in unstimulated wells (**Figure 4A**), suggesting the splenocytes unstimulated in MTB-infected mice secreted lower levels of IFN-γ. Since the numbers of splenic lymphocytes in PBS and vector control groups were not enough to be respectively used as a control for each DNA vaccine group stimulated with their self-specific proteins, five latency-related proteins were mixed and used for stimulation. The responses of PBS and Vaccae vaccine groups after hormone treatment to mixed protein were significantly higher than that to Ag85AB protein, while the vector group had no significant responses to the stimulation of mixed protein and Ag85AB protein. Compared with the mice from PBS, vector, and Vaccae vaccine groups, the number of T lymphocyte cells secreting IFN-γ in response to Ag85AB protein from mice in the *ag85ab* DNA group had a significantly increased (*P*<0.01, **Figure 4B**), indicating a significantly increased functional T cell response. There was no significant difference in the number of lymphocyte spots in response to mixed latent proteins among the 7 LTBI-associated DNA groups, and 6 LTBI-associated DNA groups except for the *rv2628* DNA group in response to mixed latent proteins also had no significant difference with the vector group (*P*>0.05). The SFCs in the Vaccae vaccine group were significantly higher than those from all other groups (*P*<0.0001, **Figure 4C**). Compared with the PBS group, the *rv1733c*, *rv1813c*, *rv2660*, and *rv2029c* DNA groups showed weaker responses to mixed proteins (excluding Rv1733c protein) (*P*<0.05 or *P*<0.01, **Figure 4B**), while the *rv2628*, *rv2659c*, and *rv3407* DNA groups showed same moderate responses to mixed proteins (*P*>0.05).

### Regulatory T cells

**Figure 5:**
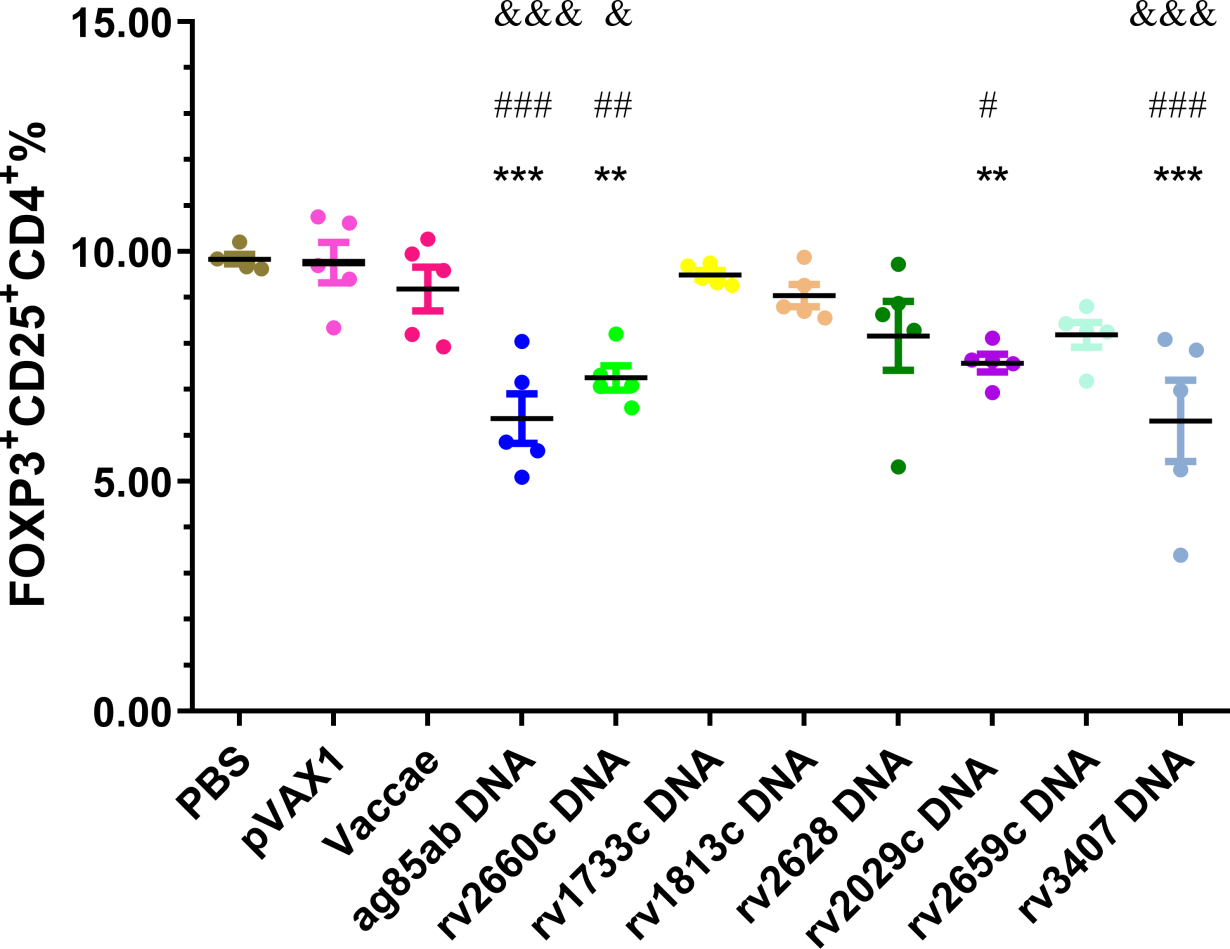
The proportion of CD4^+^CD25^+^Foxp3^+^cells in splenocytes was evaluated by flow cytometry. In comparison with PBS group: ^**^ *P* < 0.01, ^***^ *P* < 0.0001; Comparison with pVAX1 vector group: ^#^ *P* < 0.05, ^##^ *P* < 0.01, ^###^ *P* < 0.0001; Comparison with Vaccae vaccine group: ^&^ *P* < 0.05, ^&&&^ *P* < 0.0001.

Proportions of CD4^+^CD25^+^Foxp3^+^ regulatory T (Treg) cells in splenocytes of each group were determined at 3 w after stopping hydroprednisone injection (shown in **Figure 5**). The percentages of Treg cells in the *ag85ab* (*P*<0.0001), *rv2660c* (*P*<0.01), *rv2029c* (*P*<0.01, *P*<0.05), and *rv3407* DNA (*P*<0.0001) groups were significantly reduced than those of the PBS and vector groups. The percentages of Treg cells in the *ag85ab* (*P*<0.0001), *rv2660c* (*P*<0.05), and *rv3407* DNA (*P*<0.0001) groups were significantly lower than that of the Vaccae vaccine group.

### Th1/Th2/Th17 cytokines analyses

**Figure 6:**
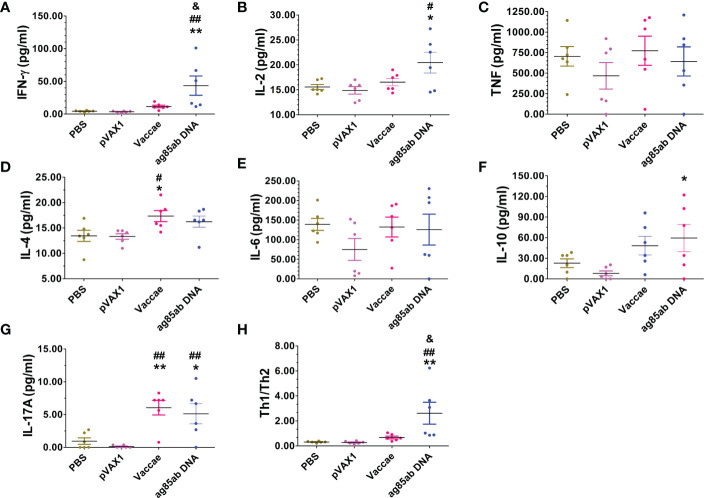
The levels of IFN-γ, IL-2, TNF-α, IL-4, IL-6, IL-10 and IL-17A cytokines in the splenocyte culture supernatant in response to recombinant Ag85AB protein for 72hrs were detected with a Mouse Th1/Th2/Th17 Cytokine Kit. **(A)** IFN-γ (pg/ml), **(B)** IL-2 (pg/ml), **(C)** TNF (pg/ml), **(D)** IL-4 (pg/ml), **(E)** IL-6 (pg/ml), **(F)** IL-10 (pg/ml), **(G)** IL-17A (pg/ml), **(H)** Th1/Th2. Compared with PBS group: ^*^ *P* < 0.05, ^**^ *P* < 0.01; Compared with vector group: ^#^ *P* < 0.05, ^##^ *P* < 0.01; Compared with Vaccae vaccine group: ^&^ *P* < 0.05.

The mouse Th1/Th2/Th17 cytokines were determined by a Mouse Th1/Th2/Th17 Cytokine Kit. The levels of IFN-γ, IL-2, and IL-17A produced by splenocytes stimulated with recombinant Ag85AB protein for 72hs in the *ag85ab* DNA group were significantly higher than those in the PBS and pVAX1 groups (*P*<0.01 or *P<*0.05, **Figure 6A**). However, compared with the pVAX1 group, the level of IL-10 in the *ag85ab* DNA group was significantly increased (*P<*0.05, **Figure 6A**). Furthermore, the levels of IL-4 (*P*<0.05) and IL-17A (*P*<0.01) in the Vaccae vaccine group were higher than those in the PBS and pVAX1 group (**Figure 6**).

**Figure 7:**
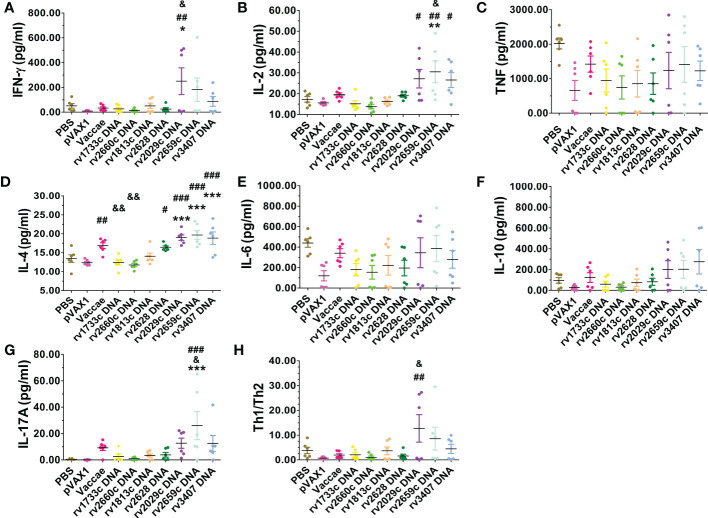
The levels of IFN-γ, IL-2, TNF-α, IL-4, IL-6, IL-10 and IL-17A cytokines in the splenocyte culture supernatant in response to recombinant mixed latency-related proteins for 72hrs were detected with a Mouse Th1/Th2/Th17 Cytokine Kit. **(A)** IFN-γ (pg/ml), **(B)** IL-2 (pg/ml), **(C)** TNF (pg/ml), **(D)** IL-4 (pg/ml), **(E)** IL-6 (pg/ml), **(F)** IL-10 (pg/ml), **(G)** IL-17A (pg/ml), **(H)** Th1/Th2. Compared with PBS group: ^*^ *P* < 0.05, ^**^ *P* < 0.01, ^***^ *P* < 0.0001; Compared with vector group: ^#^ *P* < 0.05, ^##^ *P* < 0.01, ^###^ *P* < 0.0001; Compared with Vaccae vaccine group: ^&^ *P* < 0.05, ^&&^ *P* < 0.01.

The splenocytes stimulated with recombinant mixed proteins for 72hs only in the *rv2029c*, *rv2659c*, and *rv3407* DNA vaccinated groups produced higher levels of Th1 (IFN-γ, IL-2), and Th17 (IL-17A) cytokines, and increased Th1/Th2 (**Figure 7**), but TNF-α levels among each group did not exhibit significant difference.

## Discussion

7The Cornell model is a historical mouse LTBI model, in which mice infected with *M. tuberculosis* are treated with antibiotics, resulting in no detectable bacilli by organ culture. Reactivation of infection during this culture-negative state occurred using immunosuppression. For example, Scanga CA et al. (34) reported that the mice were infected i.v. *via* the lateral tail vein with 5 × 10^3^ to 1 × 10^5^ viable bacilli for 4 weeks, treated with INH and PZA, and then treated with anti-TNF-α antibody or glucocorticoid to reactivate *M. tuberculosis*. We refer to the method to prepare the mouse LTBI model. We successfully constructed the mouse LTBI model for the first time with reference to the method reported by Scanga CA et al. (34). After the mouse LTBI model immunized by seven latency-related DNA vaccines, a previously constructed chimeric DNA vaccine with proliferating antigen (Ag85AB), and Vaccae vaccine, we injected hormones to activate the latent MTB, and evaluated the effect of these vaccines on clearing the latent MTB or preventing them reactivation from the dormant state and lay a foundation for the development of a new multi-stage vaccine that can target LTBI. Since BCG is believed to be unable to protect against LTBI (5), we did not choose BCG as a positive control to validate this model, so as not to obtain good protection.

Organ bacterial loads are one of the important indicators to evaluate protective effects on animal TB experiments (35). The less the organ colony count and the smaller the lesion range indicates that the stronger the bactericidal efficacy of the vaccine and the better the preventive efficacy. The chemotherapy could completely inhibit MTB in the lungs of infected mice for at least 13 weeks. Only the hormone treatment could activate the latent MTB, resulting in more bacteria activated and more severe lesions in the lungs of PBS and vector control groups. The chemotherapy completely killed MTB in extrapulmonary tissues (such as the livers) of infected mice, and using hormone treatment also could not reactivate them. Therefore, there was no viable bacteria growth in the liver tissues of mice in each group. Almost all the in the vaccine groups had no bacteria activated in the lungs and significantly less degree of pulmonary lesion. These results suggest that all the vaccine regimens had equal protection when measured by bacterial loads. When the degree and areas of lung pathological lesions were assessed, the potential differences among the vaccines could be observed. The degree and areas of lung pathological lesions in the *rv1733c* and *rv2659c* DNA groups were significantly reduced compared with that in the Vaccae vaccine group, indicating that these two vaccines have better protective effects on the progress and incidence of LTBI. Vaccines containing these latency-associated antigens have shown to have anti-TB effects on the TB mouse, guinea pig, rabbit, and cynomolgus macaque models (23–31). Therefore, the Vaccae vaccine, *ag85ab* DNA, and 7 latent DNA vaccines may play a role in inhibiting the proliferation and activation of latent MTB. Reece ST et al. (30) vaccinated intradermal rBCG_ureC::hly or rBCG_ureC::hly (pMPIIB01) expressing Rv2659c, Rv3407, and Rv1733c antigens in mice, and the colony counts in the lungs and spleens of mice in the rBCG_ureC::hly (pMPIIB01) group were significantly lower than those in the rBCG_ Urec:: hly group at day 200 after *M. tuberculosis* Beijing/W infection, which may be related to some MTB transition to the dormancy state. This study support that the rBCG vaccine expressing MTB latency-related antigens can improve the long-term protective efficacy against MTB. Coppola M et al. (27) immunized HLA-DR3 transgenic mice using Rv1733c synthetic long peptide combined with CpG adjuvant, which could obviously decrease the bacterial loads of the lungs from MTB-infected mice, and significantly improve the protective efficacy of BCG. Liang Y et al. (29) used *rv3407* plasmid DNA to immunize the mouse TB model, which significantly decreased the pulmonary bacterial load compared with the PBS group. These results further prove the potential of MTB latency-related antigen to improve the efficacy of the vaccine.

At present, the relationship between anti-TB protection of vaccines and immune has not been defined. However, low Th1 cellular immunity or Thl/Th2 cellular immunity imbalance is the most prominent immune characteristics in TB patients and is the major risk factor for TB (36, 37). Protective immunity against tuberculosis is largely due to a cellular immune response, in which antigen-specific functional CD4^+^ T cells and Th1/Th17 cytokines are essential for the protection against MTB (38–41). At the end of the study, after the live bacteria were eliminated and the use of hormones caused latent bacterial activation, the immune responses of the mice were evaluated. At this time, the immune responses represent the impact of various vaccines on the immune status of mice with tuberculosis recurrence. The number of T cells secreting IFN-γ in response to Ag85AB protein in the *ag85ab* DNA group had a significantly increased indicating a significantly increased functional T cell response. But the number of T cells secreting IFN-γ from 7 latency-related DNA groups didn’t significantly increase compared with the PBS group, which was consistent with our previous research (29, 31), and inconsistent with multiple studies that the MTB DosR antigens (such as Rv2029c, Rv2628) induced higher frequencies of mono- and bifunctional (IFN-γ and/or TNF-α) CD4^+^ and/or CD8^+^ T cell responses in LTBI individuals compared with pulmonary TB patients (38, 39), which may be related to many factors such as different experimental schemes, different time of antigen stimulation, and different recognition of mice on different antigens. The chemotherapy and immune-suppressing hormone used in the preparation of the mouse LTBI model of this study may have other effects on the outcome and the specific immune responses detected. The splenocytes from latency-related DNA groups stimulated by latent antigen require a long time of incubation (7 days, only one day in this study) (38, 39). In addition, the different DNA vaccines encoding latency-related proteins showed different levels of cytokine expression after the tuberculosis resurgence (29, 31, 42). Th1-type cytokines (IFN-γ and IL-2, except for TNF-α) levels in the splenic cell culture supernatant increased more than Th2-type cytokines (IL-4, IL-6 and IL-10) levels in *ag85ab*, *rv2659c*, and *rv2029c* DNA groups, indicating that Th1 cellular immune response was dominant, IFN- γ produced was important cytokines against MTB infection and IL-2 produced was important for T cell differentiation and survival, maintaining effector function and immune memory (43). Roupie V et al. (44) and Zhang W et al. (10) also showed that the mice immunized with *rv2029c*, *rv2628*, and *rv1733c* plasmid DNA could induce strong humoral and Th1-type (IFN-γ and IL-2) cellular immune response, but the *rv2628* plasmid DNA immunized mouse LTBI model in this study failed to induce the Th1 dominant immune response, which was not completely consistent with their protective efficiency. There was no significant effect on the expression of TNF-α in each vaccine group, which may be the reason why the mouse TB model is not easy to form granuloma. In addition, although TNF-α is essential to control MTB infection, its excessive production may also lead to immunopathological effects, such as hyperinflammation, caseous necrosis and cachexia, etc. (45–47). IL-17A is a member of the IL-17 receptor family, is a proinflammatory cytokine produced by the activated T cell, which mediated the production of inflammatory molecules, chemokines, antimicrobial peptides, and remodelling proteins (48), and played an important role in host defence, cells transportation, immune regulation and tissue repair (41, 49–51). In infectious diseases, IL-17A participates in the innate immune response of the host by inducing cytokines and chemokines to differentiate and migrate granulocytes. It is also involved in protective immunity against infection, participates in host immunity through cell-mediated immune response or induction of antimicrobial peptides to enhance the host’s ability to eliminate pathogens, and is a key media of host defence against infection or inflammation (41, 49, 50). Il-17 synergistically regulates IL-22, which is involved in the maintenance of intraepithelial homeostasis and repair or regeneration of epithelial cells after inflammatory injury (51). In this study, IL-17A levels in the splenic cell culture supernatant in response to Ag85AB protein in the *ag85ab* DNA and the Vaccae vaccine groups significantly increased; IL-17A levels in the splenic cell culture supernatant in response to mixed latent proteins in the *rv2659c* DNA group significantly increased than that in the Vaccae vaccine group, indicating Th17 immune response in these groups were dominant, which is consistent with the less lung pathological lesions.

Regulatory T cells are a group of lymphocytes that negatively regulate the immune response. Current studies have shown that Foxp3^+^CD4^+^CD25^+^ regulatory T cells could suppress immune response against MTB through cell-cell interactions and produced cytokines or promoted the secretion of cytokines such as IL-10 or TGF-β to down-regulated Th1 cellular immune function, which contributed to latency and proliferation of MTB (52, 53). In this study, the proportion of regulatory T cells induced by the *ag85ab*, *rv2029c*, *rv2660c* and *rv3407* DNA were significantly decreased, reducing their inhibitory effect on Th1 immune, which was conducive to improving the immune protective effect of these vaccines against MTB. The proportion of regulatory T cells in *rv2659c* DNA and Vaccae vaccine groups only showed a downward trend, there was no significant difference compared with the PBS group, which seems to suggest that there was no correlation between the number of Treg cells and protection. Unfortunately, due to the unsuccessful preparation of the Rv1733c protein in this study, the specific immunogenicity of the *rv1733c* DNA vaccine could not be fully evaluated, and the spleen lymphocytes in the *rv1733c* DNA group did not produce non-specific immune responses to mixed recombinant proteins. However, many studies have proved that Rv1733c is the most immunodominant *DosR* antigen in LTBI individuals (9, 54).

## Conclusion

MTB *ag85ab*, 7 latency-related DNA vaccines, and the Vaccae vaccine could reduce the bacterial loads in the lungs of mouse LTBI model, inhibit the resurgence of latent MTB, and lessen pathological damage. Especially *rv1733c* and *rv2659c* DNA vaccines had a higher immune preventive effect on this mouse LTBI model. Although antigen-specific Th1 and Th17 immune responses, and decreased frequencies of Treg cells were observed in some vaccine groups, there was not a clear association of these immune parameters with improved protection. Our results will help to better understand the immune characteristics and immune protective efficacy of latency-related antigens and lay the foundation for the development of a new multi-stage anti-TB vaccine.

## Data availability statement

The original contributions presented in the study are included in the article/**Supplementary Material**. Further inquiries can be directed to the corresponding author.

## Ethics statement

The animal study was reviewed and approved by The Animal Ethical Committee of the Eighth Medical Center of the Chinese PLA General Hospital.

## Author contributions

YL participated in the designing and execution of the entire experiments, conducted the statistical analysis, and wrote the manuscript. XL was involved in the whole experiments. YY prepared recombinant proteins. LX, YX and WG conducted the immunoassays. JM, LW, JW, JZ, YS, BP and XC participated in the animal experiments. YML executed the lung histopathological examination. XW and WZ designed and directed the experiments. XW revised the manuscript. All authors contributed to the article and approved the submitted version.
