# Supplementary material for: Preventive effects of Mycobacterium tuberculosis DNA vaccines on the mouse model with latent tuberculosis infection
Source: Front Immunol. 2023 Feb 13;14:1110843. doi: 10.3389/fimmu.2023.1110843 (PMC9968874; doi:10.3389/fimmu.2023.1110843)
Supplement: Supplementary file 1 [file DataSheet_1.pdf]

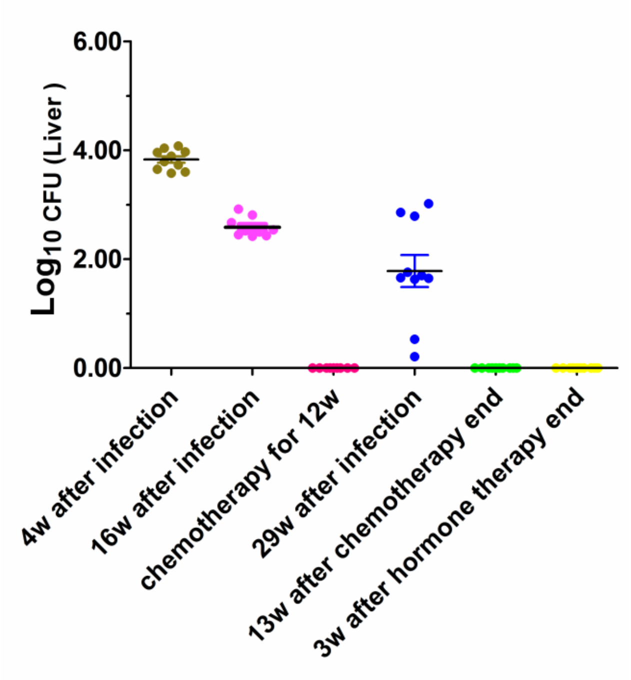

Supplementary Figure 1 The CFUs of live MTB in mouse liver were obtained from mice at different stages of mouse LTBI model preparation.

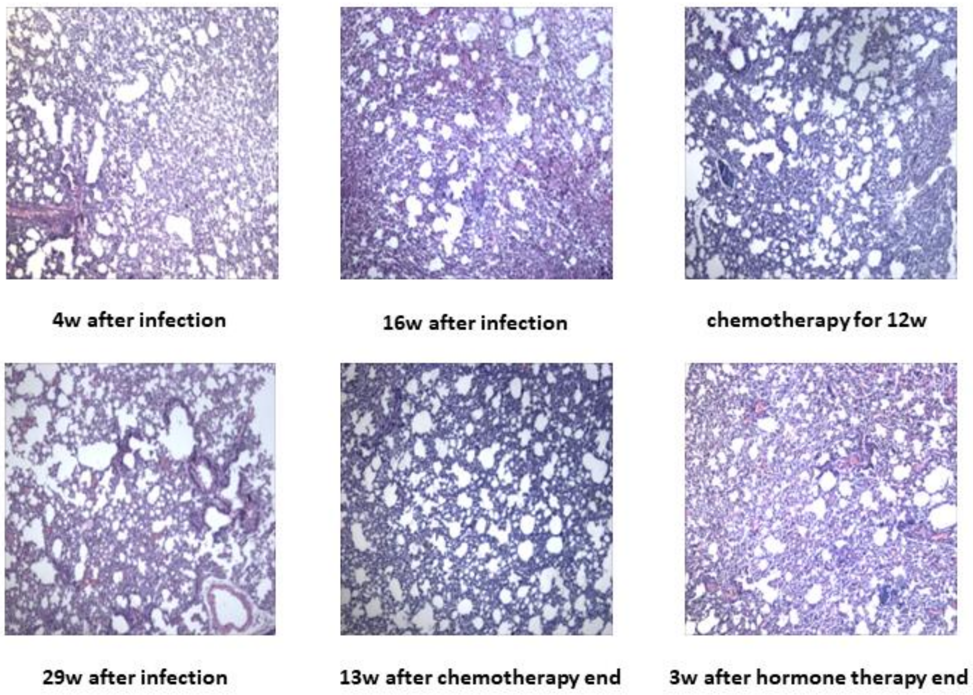

Supplementary Figure 2 Representative pulmonary histopathological photomicrographs were obtained from mice at different stages of mouse LTBI model preparation (H＆E, 40×).

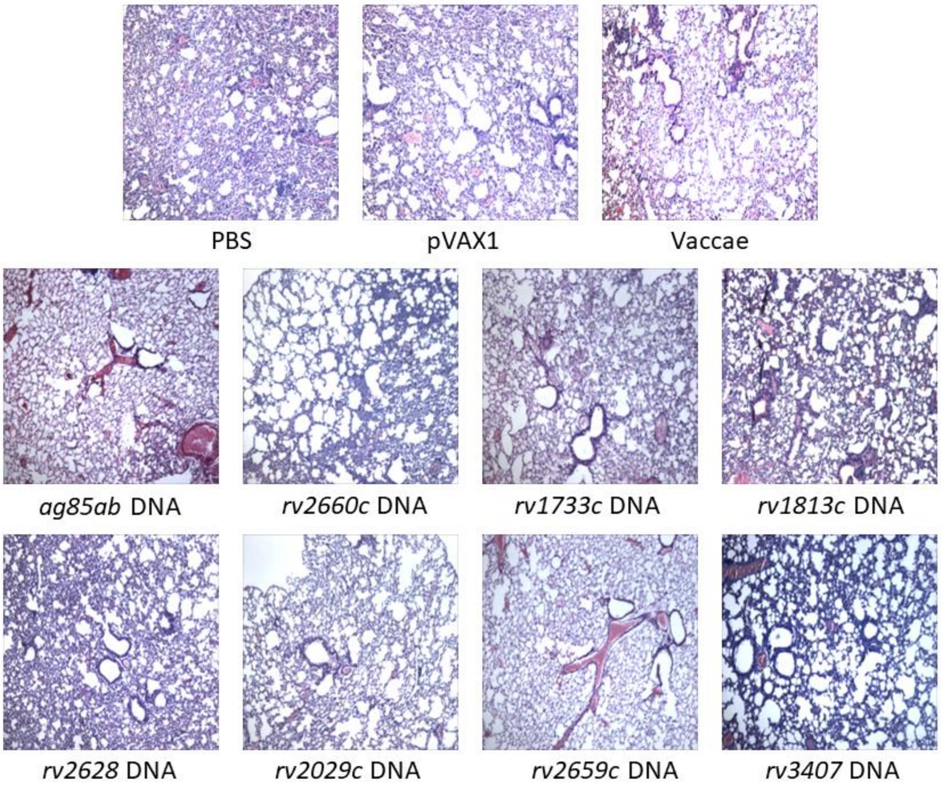

Supplementary Figure 3 Representative pulmonary histopathological photomicrographs were obtained from mice at 3 w after stopping hydroprednisone injection (H&E, 40×).
